# Exploring the perspectives of irradiated sodium alginate on molecular and physiological parameters of heavy metal stressed *Vigna radiata* L. plants

**DOI:** 10.1007/s12298-023-01286-9

**Published:** 2023-03-02

**Authors:** H. R. Moussa, Mohamed A. Taha, Eldessoky S. Dessoky, Eman Selem

**Affiliations:** 1 Radioisotope Department, Nuclear Research Center, Atomic Energy Authority, Cairo, Egypt; 2 Department of Horticulture, Faculty of Agriculture, Menoufia University, Shebin El Kom, Egypt; 3 Department of Plant Genetic Transformation, Agricultural Genetic Engineering Research Institute, Agricultural Research Center, P.O. Box, 12619, Giza, Egypt; 4 Department of Biology, College of Science, Taif University, P.O. Box 11099, Taif, 21944 Saudi Arabia; 5 Botany and Microbiology Department, Faculty of Science, Zagazig University, Zagazig, Egypt

**Keywords:** Biochemical attributes, Heavy metals, Irradiated sodium alginate, Morpho-physiological evaluation, Photosynthetic activity (^14^CO_2_-fixation), *Vigna radiata* L.

## Abstract

Heavy metal (HM) contamination causes severe detrimental effects on plant growth. Irradiated sodium alginate (ISA) has been proposed for enhancing the efficacy and selectivity of metal ion biosorption from plants under HM-toxicity. The present study has been planned to investigate the morphological, molecular, physiological, and regulation of transcript levels of defence mechanisms for alleviation of HM toxicity in *Vigna radiata* plants by irradiated sodium alginate (ISA). *V. radiata* L. plants were treated with T_0_-water (control); T_Cd_-CdCl_2_ (100 μM); T_Pb_-Pb(NO_3_)_2_ (500 μM); T_Fe_-FeSO_4_ (300 μM), and ISA solution at 75 mg/l as a foliar spray onto leaves. Our results suggested the positive impact of ISA for HM stress mitigation by *V. radiata* L. plants, through modulatory effects on molecular and physiological attributes**.** In our study, we evaluated the growth characteristics (plant height, leaf area, total fresh weight and grain weight), pigment contents, photosynthetic efficiency (^14^CO_2_-fixation), enzyme activities (nitrate reductase, ribulose-1,5-bisphosphate-carboxylase/oxygenase, and carbonic anhydrases), and macronutrient contents (P, N, and K) in metal-stressed plants. All these attributes were found to be stimulated after ISA application. Also, ISA reduced the total malondialdehyde, free proline, and total phenol content in heavy metal-exposed plants. The transcriptional expression profiling was conducted by examining the expression levels of *AtPDR12, AtATM3, AtPCR1, MPK4, MPK6,* and *AtPDR8* genes that inferred the ISA-mediated detoxification of HMs in *V. radiata* plants. The data in the present research clearly indicated that treatment with foliar sprays of ISA (75 mg/l) resulted in enhanced tolerance of *V. radiata* plants against different HM stresses.

## Introduction

Heavy metal (HM) tainting within an environment is often observed nowadays due to the majority of anthropogenic sources that adulterate the entire ecosystem along with its unpropitious effects (Saher and Siddiqui 2016). Heavy metals lead to chlorosis, necrosis, enzyme inactivation, hindered plant physiological characteristics, reduced photosynthesis, and disrupted cellular structures due to lipid peroxidation, ultimately causing cell apoptosis. This deteriorates plant growth, soil fertility, physiological alterations, overall yield, and productivities of the plants (Shahid et al. 2015). Meanwhile, HMs stimulate oxidative stress directly through reactive oxygen species (ROS) in plants and replace the essential ions to perturb the antioxidant system, biomolecule integrity, and protein structures (Choppala et al. 2014). Pb and Cd are highly toxic metals that can attach nitrogen and sulphur atoms to amino acid side chains (Thapa et al. 2012). Also, an investigation revealed that iron toxicity causes nutrient-related disorders and deficiencies of zinc, phosphorus, and calcium in the plant metabolism process and dwindles growth parameters and total yield of plants (Jaime et al. 2014). Plants have numerous ways to defend against such extreme conditions through sequestration, enzymatic transformation, efflux mechanism, chelation, etc., along with the activation of an antioxidative defence system to maintain metal homeostasis of the plants (Choppala et al. 2014). Likewise, Thapa et al. (2012) reported that, under HMs, many plants stimulate the detoxification process through effective cellular mechanisms including chelation of ions in the cytosol by peptides (phytochelatins), metallothioneins and sulfur-related compounds. Subsequently, it leads to the repair of stress-damaged essential proteins, mediates uptake or efflux pumping in the plasma membrane and compartmentalization of metals within vacuoles by *AtATM3, AtPDR12, AtPDR8* and *ZNT1* (tonoplast‐located transporters) and stimulates synthesis of mitogen-activated protein kinases (Smekalova et al. 2014). Plant defences also comprise of enzymatic and non-enzymatic systems including peroxidases, catalases, superoxide dismutase, glutathione, ascorbic acid, and tocopherol (Hossain et al. 2012). Researchers have been investigating the exploration of biomaterials in agro-systems so as to eliminate the toxicities from the ecosystem as they enter the food chain through edible organs. Sodium alginate is a naturally occurring biomaterial with a light yellow powdery or colourless crystalline natural polysaccharide of high molecular weight. The empirical formula is NaC_6_H_7_O_6_, and it is a nontoxic and highly bioactive agent that is obtained from naturally occurring brown algae Sargassum in huge quantities (Khan et al. 2010). Recently, this new trend has gained the attention of scientists who wish to utilise this natural polymer or bio-fertilizer for stimulating plant growth. Eventually, it could replace the chemical fertilizers that promote environmental pollution and facilitate the growth of many weeds. In contrast to enzymatic transformations or hydrolysis methods, gamma radiation is one of the most practical (clean one-step method), easy, economically feasible, highly safe to implement and effective technologies available to rapidly produce, in contrast to enzymatic transformations or hydrolysis methods, alginate oligosaccharides having low molecular weight and higher functional groups (El-Rehim et al. 2011; El-Mohdy 2012, 2017; Ali et al. 2014; Shabbir et al. 2017). ISA (small size oligomers) were successfully applied as natural biofertilizers (plant growth promoting substances) to induce a variety of biological and physiological processes, including the general enhancement of plant development; seed germination, shoot and root growth, flower production; to promote plant growth in a similar manner to other growth regulators; antibacterial action; reduction of heavy metal stress; and induction of phytoalexins along with inhibition of HM stresses from plants (Fahmida et al. 2013; Tariq et al. 2014; Aftab et al. 2016; Mohammad et al. 2017; Shabbir et al. 2017; Hossain et al. 2021). However, none of the investigations have been conducted so far with these crop plants in HM stress alleviation in association with ISA. Taking into consideration all the useful aspects of these oligomers (ISA) in improving plant physiology and biochemistry, a postulation was made to unravel the foliar application of ISA to improve growth, photosynthetic efficiency, productivity, and yield of *V. radiata* plants during HM toxicity under field conditions. Accordingly, the present study was carried out under natural conditions to evaluate molecular components and physiological mechanisms of plants treated by ISA under HM stressed conditions.

## Materials and methods

### Plant materials and growth conditions

Mung bean (*Vigna radiata* L.) cv. Dokki 331 seeds of uniform size were obtained from the Crop Institute, Agriculture Research Center, Egypt. Seeds were sterilised by 70% ethanol and 3.1% sodium hypochlorite (NaOCl) and then washed with deionized water. Sterilized dry seeds were germinated in pots (43 cm high × 38 cm in diameter), each filled with 13 kg of soil (sandy loam). Pots were sown with ten seeds per treatment at 3 cm depth and thinned as soon as seedlings emerged to leave five seedlings per pot (the most central healthy seedling). Mung bean plants were grown in a controlled environment growth chamber with photoperiod (14-h); photosynthetic photon flux density (450 μM m^−2^ s^−1^); day/night temperatures (21/20 °C); and relative humidity (64–76%).The fertilization of the soil was performed by adding ammonium sulphate (20% N), potassium chloride (60% K_2_O), and triple superphosphate (46% P_2_O_5_) as sources of N, P, and K, respectively. All pots were watered to reach field capacity every 5 days. A randomized complete block design was used for the experimental design. *Vigna radiata* L. plants were treated with T_0_-water (control); T_Cd_-CdCl_2_ (100 μM); T_Pb_-Pb(NO_3_)_2_ (500 μM); T_Fe_-FeSO_4_ (300 μM), and ISA solution at 75 mg/l as a foliar spray onto leaves. Gamma irradiation of SA (Sigma Aldrich Co., Missouri, USA) at a dose of 25 KGy (dose rate of 4.70 KGy/h) was performed by using a ^60^Co source of radiation at the Atomic Energy Authority, National Center for Radiation Research and Technology, Egypt. After 10 days of seedling emergence, foliar spray with ISA solution was applied twice a week on all the plant in the pots during morning hours using manual pump. The measurements of the growth characteristics, plant height (cm), fresh weight (g) and leaf area (cm^2^), and determination of the biochemical analysis were performed 40 days after sowing. After 80 days, the pods were harvested and dry weights of 100 grains (g) were recorded.

### Lethal toxicity of heavy metals

On the basis of acute LC50 values, the level of heavy metal toxicity was calculated (data not included in the text). In each germination test, eight heavy metal concentrations (CdCl_2_; Pb(NO_3_)_2_ and FeSO_4_) were used, with three samples per test.100 seeds were used, and they were distributed on double-layered filter papers (3 mm, Whatman No. 1), which were then placed in 12-cm Petri dishes and moistened with either 10 ml of double-distilled water (ddH_2_O) or 10 ml of metal solutions. The seeds were not pre-soaked or sterilized. The Petri dishes were covered and incubated at 22 ± 2 °C. Once the seed coat was broken, the seeds were scored as having germinated. Inhibition of germination (%) was calculated in comparison to the control samples and the LC50 values were calculated using methods described by Niewiada et al. (2018).

### Biochemical and chemical analysis

Photosynthetic pigment contents were analyzed in fresh leaves by the procedure of Lichtenthaler and Buschmann (2001). The photosynthetic pigments, thus measured, were expressed as mg g^−1^ FW. The photosynthetic efficiency (^14^CO_2_-fixation) was estimated in the Radioisotope Department, Atomic Energy Authority, Egypt (Moussa and Mohamed 2016) in fresh leaves and determined as 10^3^ Becquerel mg^−1^FW. The free proline level (μg g^−1^FW) was determined in fresh leaves and roots according to Bates et al. (1973). A total phenol level (μg g^−1^DW) was estimated in dry leaves and roots according to Sadasivam and Manickam (2008). Lipid peroxidation (malondialdehyde, MDA) content was determined in fresh leaves and roots (μM g^−1^FW) by the method of Chen et al. (2013). The activity of nitrate reductase (nM NO_2_ g^−1^ FW h^−1^) was determined in fresh leaves and roots according to the method of Jaworski (1971). The enzyme Ribulose-1,5-bisphosphate-carboxylase/oxygenase (RuBPCase, EC 4.1.1.39) was measured in fresh leaves and roots (mgg^−1^ FW) using the Warren et al. (2000) method. The activity of carbonic anhydrase (μMCO_2_ kg^−1^ FW S^−1^) in fresh leaves and roots was measured following the procedure of Dwivedi and Randhawa (1974). Macronutrients were evaluated in dry roots and leaves (N, P and K) were determined (mg g^−1^DW) by the method of Cottenie et al. (1982).

### Germination test

**Fig. 1:**
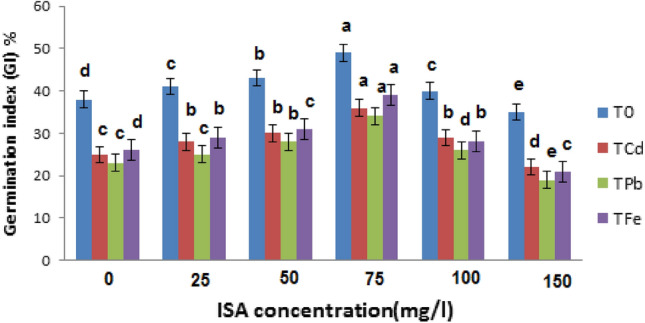
The germination index (GI) of *V. radiata* L. seeds treated with different concentrations of ISA in response to heavy metal stresses Means within a column followed by the same letter(s) are not significantly different (*p* ≤ 0.05). Error bars (┬) show SE

The germination test result was used to define the ISA treatment of seedlings, 100 seeds were treated with different concentrations of ISA solution (0.0, 25, 50, 75, 100 and 150 mg/l) and subjected to heavy metal stress treatments: **T**_**0**_**-**water (control); **T**_**Cd**_**-**CdCl_2_ (100 μM); **T**_**Pb**_**-**Pb(NO_3_)_2_ (500 μM) and **T**_**Fe**_**-**FeSO_4_ (300 μM) were grown on solid 0.5-strength Murashige and Skoog (MS) medium for seven days (Fig. 1). When the radical reaches 0.3 cm, the number of germinated seeds is recorded daily for seven days to calculate the germination index.

The germination index (GI) was calculated using the following equation:

$$ {\text{GI }} = {\text{ N}}_{{1}} /{1 } + {\text{ N}}_{{2}} /{2 } + {\text{ N}}_{{3}} /{3}......,{\text{N}}_{{\text{n}}} /{\text{n}} $$

where N_1_, N_2_, N_3_, ……, N_n_ means the germinated seeds number on 1, 2, 3,……,n days.

### Real-time quantitative RT-PCR

Forty-day-old *V. radiata* L. seedlings (stems and leaves) of the control, heavy metal stresses and ISA treatments were used for extraction and purification of high quality, pure, and intact total RNA. Stems and leaves were washed with tap water, then sterilized with 70% ethanol for 40 s, and then rinsed twice with sterile water. Subsequently, plant organs were placed on sterile filter paper to remove water and finally ground rapidly in liquid nitrogen to obtain a fine powder for RNA extraction. A super Script III reverse transcriptase was used for cDNA-synthesis. Real-Time Quantitative PCR was performed by Eppendorf Mastercycler EP-Realplex machine (Eppendorf, Germany). All of the reactions were conducted in triplicate simultaneously. A final volume of 20 µl of the reaction mixture is formed by mixing ten micro liter of 2 × SYBR® Premix Ex Taq™ (Otsu, Japan, Takara Bio, Inc.), 1 µl of reverse transcription-polymerase chain reaction, and 200 nM of gene-specific reverse transcription primers. All polymerase chain reactions was achieved by using this thermal profile: pre-denaturation at 94 °C for two minutes with subsequent 40 cycles of 16 s (96 °C) for denaturation, 16 s (57 °C) for annealing, and 21 s (71 °C) for extension. The collected data was analysed using the comparative C_t_ (2^_ΔΔCt^) procedure (Schmittgen and Livak 2008). Primer sequences used for qRT-PCR are given in Table 1.

**Table 1:** Primers and accession numbers for genes involved in quantitative real-time PCR (qPCR)

Accession number	Gene	Oligo sequence	
At1g15520	*AtPDR12*	Forward reverse	5'- TATCGGTGAGATGACTGTTCGTG -3′ 5'- TTCACCTGCTGTTGACATCGC -3'
At5g58270	*AtATM3*	Forward reverse	5'- TGCTTTTGCCTGGATTACTTCTC -3′ 5'- TTCTAAACTTGGTTCGCCACTGT -3'
At1g14880	*AtPCR1*	Forward reverse	5'- TTTGCTGTAACCTCTGTGCTTTGA -3′ 5'- CCATCGCCACTCCACCTTG -3'
At4g01370	*MPK4*	Forward reverse	5'-ACATGTCGGCTGGTGCAGT-3′ 5'-AATATGGGTGGCACAACGC-3'
At2g43790	*MPK6*	Forward reverse	5'-TAAGTTCCCGACAGTGCATCC-3′ 5'-GATGGGCCAATGCGTCTAA-3'
At1g59870	*AtPDR8*	Forward reverse	5'- GAGAGGTCGCAATGGGGAGA -3′ 5'- GTCTCTCATTTCCCCAGGCAT -3'
At2g01010	*18S rRNA*	Forward reverse	5'-CGGCTACCACATCCAAGGAA-3′ 5'-TGTCACTACCTCCCCGTGTCA-3'

### Statistical data analysis

The statistical analysis was performed on all the data. The means were separated using Fisher's unprotected Least Significant Difference (LSD) at the 5% level of significance (Gomez and Gomez 1992).

## Results

### Impact of ISA on seed germination under heavy metals stresses

The germination index (GI) of plants treated with HMs was significantly reduced by 34.21, 39.47, 31.57% as compared to control plants. While the treatment of ISA (75 mg/l) led to improved GI of Cd, Pb, and Fe stressed plants by 44, 47.8, and 50%, in contrast to plants with metal-alone treatments (Fig. 1). The maximum percentage of the germination index (GI) was obtained with 75 mg/l of ISA. The control plants treated with ISA (75 mg/l) showed better GI than the control plants without ISA treatment. Hence, ISA in general aids in germination efficiency of the plant. The GI of seeds treated with 100 mg/l of ISA was slightly decreased. Meanwhile, it was considerably decreased at 150 mg/l of ISA (Fig. 1). As a result, exogenous application of ISA at a concentration of 75 mg/l induced the maximum germination potential. Therefore, ISA solution (75 mg/l) was further used for the subsequent analysis in the present investigation.

### Plant growth parameters

The present study revealed that HMs (Cd, Pb, Fe) stressed *V. radiata* L. showed a significant decrease in plant growth parameters in terms of plant height, leaf area, total fresh weight, and weight of 100 grains. A decline of 13.46, 21.1, 11.53% in plant height, 9.79, 11.43, 8.91% in leaf area, 19.53, 25.1, 18.35% in total fresh weight, and 37.5, 31.12, 18.75% weight of 100 grains was observed, respectively, in Cd, Pb, and Fe exposed plants relative to control plants. After the exogenous treatment of ISA solution (75 mg/l), substantial enhancement of plant height (11.11, 14.63, 10.86%), leaf area (2.92, 3.82, 2.89%), total fresh weight (9.15, 14.5, 9.28%), and weight of 100 grain (20, 18.18, 15.38%) was found in Cd, Pb, and Fe stressed mung plants compared to only metal stressed plants. All these growth attributes were triggered in control as well as ISA treated HM exposed plants (Table 2).

**Table 2:** Impact of ISA on plant growth parameters (plant height, leaf area, total fresh weight, and weight of 100 grain) of heavy metal-stressed *V. radiata* L. plants

Treatments	ISA (mg/l)	Plant height (cm)	Leaf area (cm^2^)	Total fresh weight (g)	Weight of 100 grain (g)
**T**_**0**_	**0.0** **75.0**	52 ± 1.6^b^ 61 ± 2.4^a^	796 ± 39.8^b^ 836 ± 41.8^a^	93.7 ± 4.7^b^ 120.6 ± 7.2^a^	16 ± 0.48^b^ 28 ± 0.84^a^
**T**_**Cd**_	**0.0** **75.0**	45 ± 1.3^d^ 50 ± 2.5^b^	718 ± 21.5^e^ 739 ± 36.9^c^	75.4 ± 2.3^e^ 82.3 ± 5.6^c^	10 ± 0.51^e^ 12 ± 0.48^d^
**T**_**Pb**_	**0.0** **75.0**	41 ± 2.5^e^ 47 ± 3.5^c^	705 ± 49.3^f^ 729 ± 36.4^d^	70.1 ± 2.1^f^ 80.3 ± 3.2^d^	11 ± 0.32^e^ 13 ± 0.41^d^
**T**_**Fe**_	**0.0** **75.0**	46 ± 3.2^c^ 51 ± 2.0^b^	725 ± 21.7^d^ 746 ± 44.8^c^	76.5 ± 1.5^e^ 83.6 ± 2.5^c^	13 ± 0.44^d^ 15 ± 0.53^c^

Means ± standard error of three variables. Number with the same letter in each column are not significantly different at *P* < 0.05

Number with the same letter in each column are not significantly different at *P* < 0.05

### Physiological and biochemical parameters

### Photosynthetic pigments (total chlorophyll and carotenoids) and photosynthetic efficiency (^14^CO_2_-fixation)

Our study illustrated that heavy metal-stressed *V. radiata* L. inhibited pigment content (total chlorophyll and carotenoids) as well as photosynthetic activities (^14^CO_2_-fixation) of the plants. A decline in the total chlorophyll content of HMs was found by 29.22% (Cd), 31.16% (Pb), and 33.33% (Fe). Furthermore, after foliar spraying with ISA (75 mg/l), total chlorophyll contents increased by 27.52, 27.35, and 28.24% in Cd, Pb, and Fe stressed plants, respectively. Moreover, the reduction in the carotenoid level was found to be 32.82% (Cd), 25.12% (Pb), and 25.64% (Fe) as compared to untreated plants, after which their levels were stimulated by ISA by 19.08, 10.95, 24.13%, respectively. Apart from this, the photosynthetic efficiency (^14^CO_2_-fixation) of mung plants was also reduced in plants after metal treatment by 23.31% in Cd, 31.28% in Pb, and 11.17% in Fe as compared to untreated plants. An aggravation in their levels was further found after the application of ISA. A stimulation of 16.8, 22.32, and 9.62% was observed in ISA-treated Cd, Pb, and Fe plants relative to metal-alone raised plants. The inclined photosynthetic pigment concentration and photosynthetic activity compared to HMs stressed or control mung bean plants have been tabulated in Table 3.

**Table 3:** Impact of ISA on chlorophylls *a* + *b* (mg g^−1^FW), carotenoids (mg g^−1^FW), photosynthetic efficiency (10^3^ Becquerel mg^−1^FW), total phenols (μg g^−1^DW), free proline (μg g^−1^FW), and MDA (μM g^−1^FW) in heavy metal- stressed *V. radiata* L. plants

Treatments	ISA (mg/l)	Chlorophyll (a + b)	Carotenoids	Photosynthetic activity	Total phenol	Free proline	MDA
T_0_	**0.0** **75**	4.62 ± 0.14^b^ 6.12 ± 0.25^a^	1.95 ± 0.09^b^ 2.89 ± 0.06^a^	16.3 ± 0.65^b^ 24.1 ± 0.72^a^	7500 ± 675^e^ 6680 ± 400^f^	42.9 ± 1.3^e^ 36.8 ± 2.2^f^	11.9 ± 0.7^f^ 10.1 ± 0.4^f^
T_Cd_	**0.0** **75**	3.27 ± 0.11^e^ 4.17 ± 0.21^c^	1.31 ± 0.05^ g^ 1.56 ± 0.08^e^	12.5 ± 0.37^e^ 14.6 ± 0.25^c^	8360 ± 501^b^ 7850 ± 392^d^	56.3 ± 3.4^b^ 50.0 ± 2.0^d^	22.7 ± 2.5^b^ 16.3 ± 1.4^d^
T_Pb_	**0.0** **75**	3.18 ± 0.06^f^ 4.05 ± 0.20^d^	1.46 ± 0.07^f^ 1.62 ± 0.09^d^	11.2 ± 0.18^f^ 13.7 ± 0.17^d^	8500 ± 425^a^ 8090 ± 647^c^	58.0 ± 3.1^a^ 53.9 ± 2.5^c^	25.7 ± 2.5^a^ 17.8 ± 0.6^d^
T_Fe_	**0.0** **75**	3.08 ± 0.12^ g^ 3.95 ± 0.18^d^	1.45 ± 0.05^f^ 1.80 ± 0.06^c^	13.5 ± 0.19^d^ 14.8 ± 0.22^c^	8000 ± 560^c^ 7700 ± 680^d^	51.4 ± 3.7^d^ 46.8 ± 2.4^e^	20.1 ± 1.2^c^ 14.0 ± 0.7^e^

Means ± standard error of three variables. Number with the same letter in each column are not significantly different at *P* < 0.05

Number with the same letter in each column are not significantly different at *P* < 0.05

### Total phenols, free proline and malondialdehyde content

While evaluating total phenols, we found that the content was enhanced in HM stressed plants by 17.51, 21.10 and 15.26% in Cd, Pb and Fe stressed plants. In HM stressed mung plants, the addition of ISA increased their quantities by 6.49, 5.06, and 3.89%, respectively, when compared to metal-alone control plants. A similar observation was gained in the case of total proline content where Cd, Pb, and Fe stimulated the proline content, by 35.86, 46.46, and 35.32% in contrast to control plants. After treatment with ISA, their levels were aggravated by 12.6, 7.6, and 3.21%, respectively. Also, the MDA content was found to be triggered in plants exposed to metal toxicity (61.38% (Cd), 76.23% (Pb), and 38.61% (Fe)) compared to control plants. A further rise in their content was observed in plants after ISA treatment by 39.26% (Cd), 44.38% (Pb), and 43.57% (Fe), respectively, as compared to metal control plants.

### Ribulose-1,5-bisphosphate-carboxylase/oxygenase, carbonic anhydrase and nitrate reductase activities

**Fig. 2:**
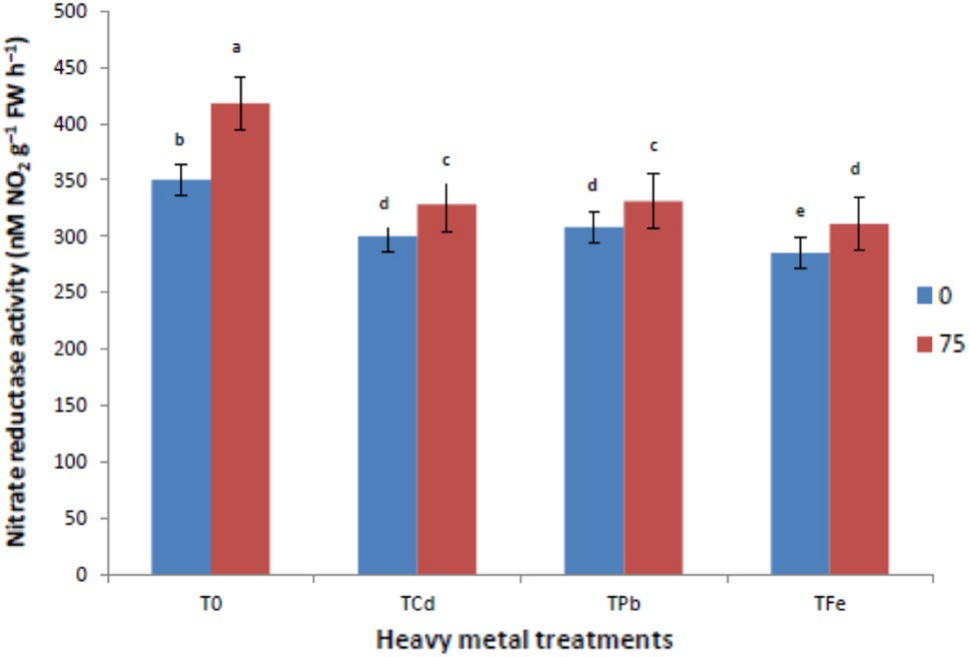
Effect of ISA on nitrate reductase activities in heavy metal-stressed *V. radiata* L. plants. Means within a column followed by the same letter(s) are not significantly different (*p* ≤ 0.05). Error bars (┬) show SE

**Fig. 3:**
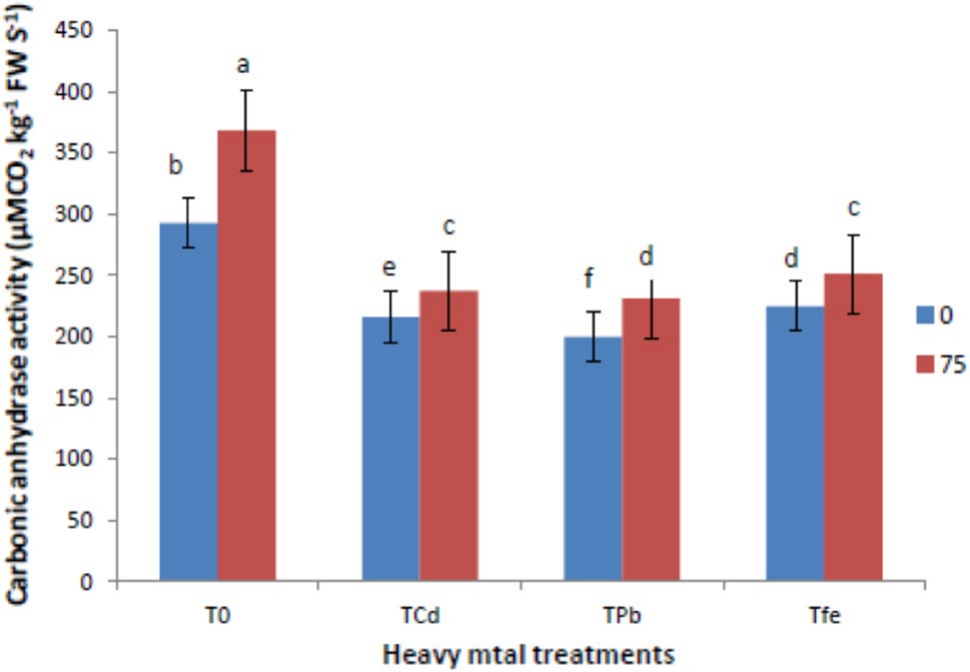
Effect of ISA on carbonic anhydrase activities in heavy metal- stressed *V. radiata* L. plants. Means within a column followed by the same letter(s) are not significantly different (*p* ≤ 0.05). Error bars (┬) show SE

**Fig. 4:**
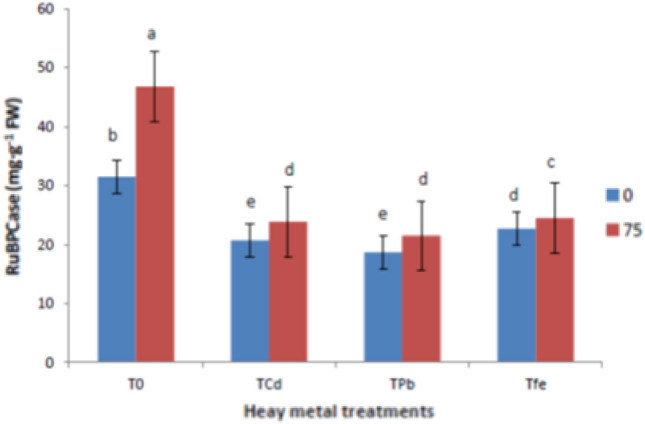
Effect of ISA on RuBPCase activities in heavy metal-stressed *V. radiata* L. plants. Means within a column followed by the same letter(s) are not significantly different (*p* ≤ 0.05). Error bars (┬) show SE

HM stressed *V. radiata* plants, revealed a significant reduction of the enzyme activities of nitrate reductase, carbonic anhydrase, and RuBPCase (Figs. 2, 3 and 4). A decline in the activity of ribulose-1,5-bisphosphate-carboxylase/oxygenase by 34.28, 40.63 and 27.93% was observed in plants subjected to Cd, Pb, and Fe toxicity as compared to control plants. ISA treatment increased these activities by 15.45, 14.97, and 7.92% compared to metal control plants. A similar trend was observed for carbonic anhydrase where a decrease of 26.27, 31.74, and 23.20% was observed in plants subjected to Cd, Pb, and Fe toxicity as compared to control plants. Further, its activity got enhanced by 9.72, 15.5, and 11.55% after ISA treatment in metal-exposed plants. Likewise, nitrate reductases also showed a reduction of 14.28, 12%, and 18.57 in plants subjected to Cd, Pb, and Fe toxicity as compared to control plants. The foliar application of ISA induced these activities by 9.33, 7.46 and 9.12% in metal raised plants. Therefore, the foliar treatment with ISA (75 mg/l) enhanced all these enzyme activities.

### Mineral nutrition (N, P and K)

**Fig. 5:**
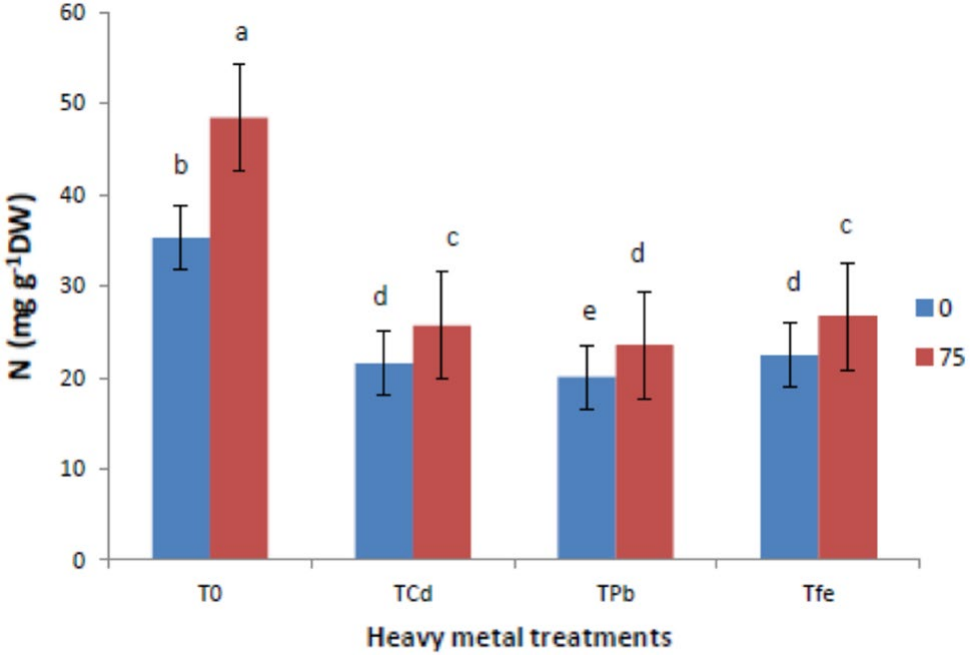
Effect of ISA on nitrogen contents in heavy metal-stressed *V. radiata* L. plants. Means within a column followed by the same letter(s) are not significantly different (*p* ≤ 0.05). Error bars (┬) show SE

**Fig. 6:**
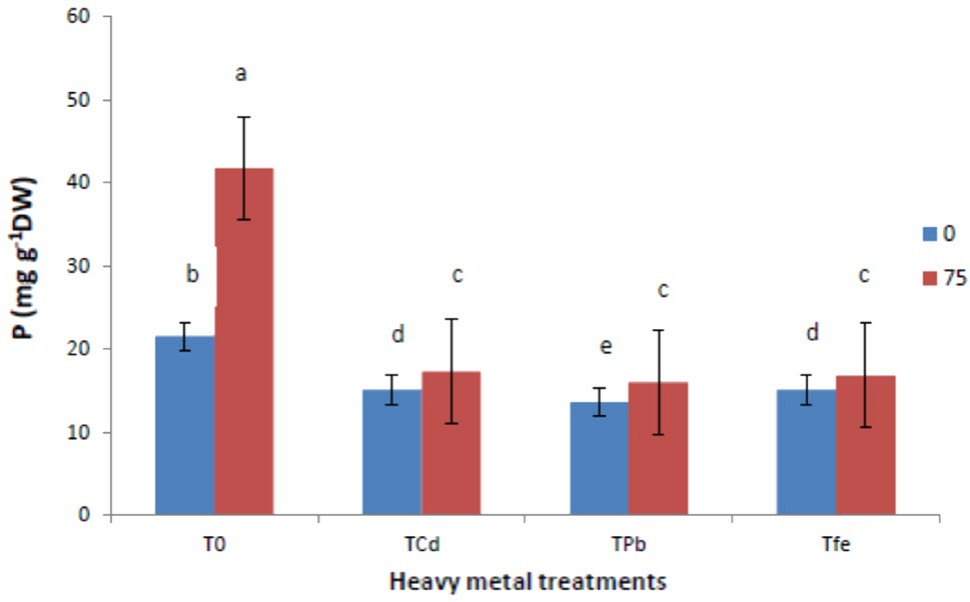
Effect of ISA on phosphorus contents in heavy metal-stressed *V. radiata* L. plants. Means within a column followed by the same letter(s) are not significantly different (*p* ≤ 0.05). Error bars (┬) show SE

**Fig. 7:**
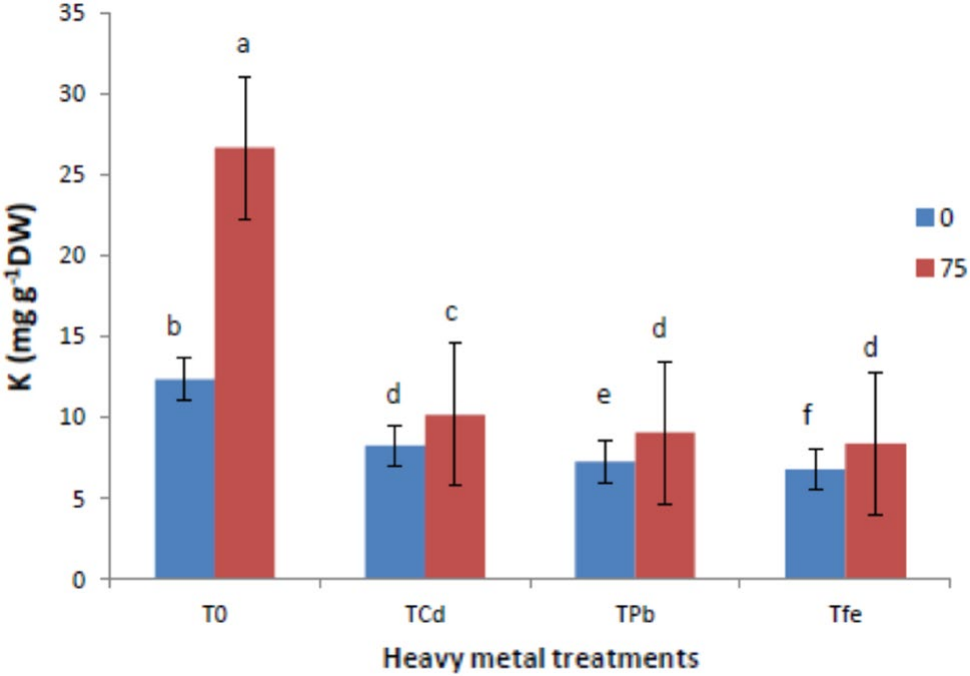
Effect of ISA on potassium contents in heavy metal-stressed *V. radiata* L. plants. Means within a column followed by the same letter(s) are not significantly different (*p* ≤ 0.05). Error bars (┬) show SE

Figures 5, 6 and 7 depicts results encompassing macronutrient levels of N, P and K under the influence of HMs and ISA treatment. It was reported that HM stressed *V. radiata* L. showed reduction in the mineral nutrition (N, P and K) under Cd toxicity by (38.81, 29.76 and 33.06%), Pb toxicity by (43.05, 36.74 and 41.12%) and Fe toxicity by (36.26, 29.76 and 45.16%), respectively, compared to control plants. However, foliar treatment with ISA solution (75 mg/l) significantly enhanced the macronutrient content. An induction in the N content was observed in ISA treated HMs raised plants, that is, Cd by 18.98%, Pb by 17.41% and Fe by 18.66%. Moreover, P and K content were also triggered in metal-exposed plants after ISA application by 14.56% (Cd), 17.64% (Pb) and 11.25% (Fe) for P and 22.89% (Cd), 24.65% (Pb) and 25% (Fe) for K, respectively.

### Gene expression profiling

Gene expression profiling studies of *V. radiata* L. plants under the exposure of Pb, Cd, and Fe along with foliar ISA (75 mg/l) applications have been conducted and tabulated in Table 4. The possible role of ISA in regulating transcript levels of genes under heavy metal stress has been elucidated. Our results showed that ISA foliar spray modulated the expression levels of genes *AtPDR8, AtPDR12, AtATM3, AtPCR1, MPK4* and *MPK6* in HMs (Cd, Pb, and Fe) stressed plants. The obtained data showed that exogenous application of ISA (75 mg/l) modulated *AtPDR8, AtPDR12,* and *AtATM3* transcript levels by 46.86, 44.39, and 37.56% in Cd, by 74.63, 40.42, and 8.73%, in Pb and by 47.41%, 35.67%, and 16.55%, in Fe stressed plants in contrast to metal control plants. In addition, the expression levels of *AtPCR1, MPK4* and *MPK6* genes were also modulated by 33.85, 1.45 and 5.21% in Cd, by 34.37, 7.75 and 0.76% in Pb, and by 36.13, 0.72 and 7.05% in Fe-stressed plants compared to metal-alone treated plants.

**Table 4:** The selected genes and their expression values related to Cd, Pb, and Fe tolerance of *V. radiata* L. plants treated with ISA (75 mg/l)

Gene	Relative gene expression					
	CdCl_2_		Pb(NO_3_)_2_		FeSO_4_	
	ISA	0.0	ISA	0.0	ISA	0.0
AtPDR8	2.39 ± 0.05^b^	1.27 ± 0.04^c^	4.14 ± 0.08^a^	1.05 ± 0.04^d^	2.51 ± 0.08^b^	1.32 ± 0.03^e^
AtPDR12	2.23 ± 0.06^a^	1.24 ± 0.03^d^	1.88 ± 0.07^b^	1.12 ± 0.04^e^	1.71 ± 0.02^c^	1.10 ± 0.02^e^
AtATM3	1.89 ± 0.03^a^	1.18 ± 0.01^c^	1.26 ± 0.02^b^	1.15 ± 0.05^c^	1.51 ± 0.03^b^	1.26 ± 0.02^c^
AtPCR1	1.92 ± 0.04^c^	1.27 ± 0.02^f^	2.56 ± 0.04^a^	1.68 ± 0.04^d^	2.38 ± 0.07^b^	1.52 ± 0.06^e^
MPK4	1.37 ± 0.03^a^	1.39 ± 0.02^a^	1.16 ± 0.05^b^	1.25 ± 0.06^b^	1.40 ± 0.04^a^	1.41 ± 0.05^a^
MPK6	1.15 ± 0.03^d^	1.21 ± 0.03^d^	1.33 ± 0.03^c^	1.32 ± 0.07^c^	1.56 ± 0.02^b^	1.45 ± 0.06^b^

Means ± standard error of three variables. Number with the same letter in each column are not significantly different at *P* < 0.05

## Discussion

The present study revealed that HMs (Cd, Pb, and Fe) reduced the GI of *V. radiata* plants. Heavy metals hampered cell proliferation, seed germination, root growth, and plant architecture in a variety of plant species (Moussa and Sabah 2010; Lin and Aarts 2012). Carrageenan and chitosan (natural polysaccharides), along with ISA also induce growth parameters and other related functions in plants (Aftab et al. 2011; Adeeba et al. 2011; Tariq et al. 2014; Mohammad et al. 2017). ISA, treatment induced a stimulatory effect on morphological characteristics, plant growth and total yield of plants (Naeem et al. 2012a, b; Aftab et al. 2016; Taha and Sorour 2019). Theoretically, plants may be able to identify particular natural polysaccharide oligomers, which would further encourage plant growth, development, shoot elongation, and defensive response (El-Mohdy 2017). The present study also confirmed the hindrance of the morphological attributes in HM stressed plants in terms of plant height, leaf area, total fresh weight and weight of 100 grain. For instance, HMs (As, Cd, Cu, Cr, and Fe) have been known to cause disturbed plant growth in Brassica (Silva et al. 2016; Du et al. 2020). We also speculated that the ISA foliar application improved the morphological traits of the metal-stressed plants. Depolymerized ISA significantly increased growth parameters, CO_2_ assimilation and net photosynthesis in *Oryza sativa* (Hien et al. 2000). Many of the researchers investigated that ISA-treatment in plants could act as a practicable biofertilizers to stimulate growth parameters, especially plant elongation (shoot and root) and, in turn, induce an improvement in physiological parameters like alkaloid content, total plant yield, and productivities of agricultural crops (Talaat and Abdallah 2011; Naeem et al. 2012a, b; Naeem et al. 2015; Mohammad et al. 2017). Along with this, *Trigonella foenum-graecum* L. plants showed higher biomass, leaf area, plant height, root and shoot length, and leaf number after ISA treatments (Battah et al. 2021). ISA might act as a growth promoter and biofertilizer for boosting the growth and other functions of plants (Dar et al. 2016). The main mechanism behind the ISA-mediated seed germination rate is associated with its biosorbent nature to capsulate the metal ions so as to prevent their harmful impact on plants and their metabolic actions. Additionally, it was noted that depolymerized polysaccharides including alginate, carrageenan, and chitosan have unique features such as promoting germination and shoot elongation (Idrees et al. 2011; Taha and Sorour 2019; Mirajkar et al. 2019). Photosynthesis was promoted by the fact that smaller alginate molecules exhibited better stomatal absorption (Hossain et al. 2021). The usage of ISA may have boosted photosynthate's efficient assimilation and translocation, which may have contributed to the spearmint plant's improved leaf growth (Sadiq et al. 2017). In contrast to untreated plants, the absorption of ISA acted as a growth promoter, causing plant root and shoot elongation, boosting plant yield, and improving physiological parameters (El-Rehim et al. 2009). Degraded alginate plays a crucial role in triggering cell signaling in various plants, which in turn stimulates several physiological processes and accelerates plant growth (Hien et al. 2000). ISA treatment increasing the area of the leaves lead to an increase in CO_2_ fixation, and therefore an increase in photosynthesis (Naeem et al. 2012a, b; Taha and Sorour 2019). Application of irradiated sodium alginate to various crops induces growth enhancements (Albayrak 2005; Chmielewskia et al. 2007; Naeem et el. 2015; Aftab et el. 2016; Uddin et al. 2020). ISA (alginate oligomers) application at 120 mg/l played an important role of cell signalling for the induction of phytoalexins which increased growth parameters and productivity in tea (Sadiq et al. 2017). The present study also showed that HM stresses hindered photosynthetic efficiencies (^14^CO_2_-fixation) and pigments (chlorophyll and carotenoids) in plants (Delmail et al. 2011; Tripathi et al. 2012; Talukdar 2013). The use of ISA increased photosynthetic pigment content, which could be attributed to increased chlorophyll biosynthesis, chloroplast size and number, and overall grana development (Papia et al. 2017). Furthermore, increased nitrogen (a constituent of chlorophyll molecules) absorption by ISA treatment was positively related to the synthesis of active photosynthetic pigment content (Khan et al. 2010). The ISA application protected chloroplast structures from outside attack in order to maintain the proper functioning of the plant processes (Dar et al. 2016). The higher photosynthetic rate brought by the foliar application of ISA may have been caused by the increased content of photosynthetic pigments, stomatal conductance, enhanced Rubisco and CO_2_ fixation which in turn increased phosphorylation processes (Aftab et al. 2011, 2016; Khan et al. 2011; Sarfaraz et al. 2011). In addition, we have also revealed that total phenols, free proline and MDA content of plants were enhanced after metal treatment due to an increase in ROS (Rizvi et al. 2020). Exogenous treatment with ISA significantly decreased total phenols, free proline, and MDA content as compared to the control plants (Naeem et al. 2011). ISA could also stimulate the antioxidant enzymes SOD, CAT, and PPO, whose combined purpose is to protect cells from oxidative damage (Papia et al. 2017). A further study was conducted to study the effects of HMs and ISA on enzyme activities of nitrate reductase, carbonic anhydrase, and RuBPCase of *V. radiata* plants. However, our study proved that ISA application increased P and N levels that would boost nitrate concentration and nitrate reductase activity of many plants, which is consistent with the results of Naeem et al. (2011); Aftab et al. (2011); Aftab et al. (2016); Shabbir et al. (2017); Jaleel et al. (2017) and Battah et al. (2021). Increased nitrate levels in plant tissues stimulate functional nitrate reductase by increasing levels of nitrate-sensing essential proteins (Campbell 2002). Carbonic anydrases are Zn-comprising enzymes that play vital roles in plant photosynthetic processes and reversibly catalyse CO_2_ into carbonic acid, followed by stimulating CO_2_ concentrations for Rubisco in photosynthetic processes (Shabbir et al. 2017). In addition, carbonic anhydrase activities increased after ISA foliar spray, which has been profoundly effective in cell-signaling and concerned physiological processes in plants (Naeem et al. 2011; Idrees et al. 2012; Aftab et al. 2016; Jaleel et al. 2017; Shabbir et al. 2017). Depolymerized polysaccharide (ISA) not only induces stomatal conductance but also facilitates CO_2_ diffusion through stomatal pores, thereby increasing the activity of carbonic anhydrases (Naeem et al. 2011). In particular, carbon fixation is directly linked to carboxylases as well as oxygenase activities of Rubisco. Henceforth, ISA activities towards carbonic anyhydrase reflect the combinatorial effects of associated factors and also in inducing the Rubisco activities. The most probable mechanism of carbonic anhydrase elevation in the present study might be due to ISA-induced de novo synthesis of carbonic anhydrases involved in transcriptional and translational processes. This in turn would mediate CO_2_ fixation and is expected to further elevate the fresh and dry weights of plants. Furthermore, the mineral nutrition status was also determined in the terms of N, P and K and found to be lowered in metal stressed plants (Bejarano et al. 2021).

Our findings also revealed that ISA induced an increase in nutrient uptake (N, P, and K) in metal-stressed plants. Previous studies conducted by Khan et al. (2007) ascribed the important role of ISA in increasing water and mineral uptake from the soils, followed by an increased translocation rate of photoassimilates and many active metabolites to the sink tissues along with improved dry matter and total yield production of the plants. It has been revealed that exogenous application of ISA might act as extracellular signalling molecules to stimulate plant growth and via induction of tolerance mechanisms in plants through initiating biosynthesis of many enzymes, including the activators of gene expression (Papia et al. 2017). ISA treatment increased the synthesis and translocation rate of carbon compounds to the sink tissues, increasing the total yield (Papia et al. 2017). Additionally, ISA increased the absorption efficiency and utilization of N, P, and K, which further led to raising the productivity and yield of the plants (Naeem et al. 2012a, b; Tariq et al. 2014; Mohammad et al. 2017). Presumably, the ISA induces the membrane permeability in a similar fashion to that of growth regulators (Idrees et al. 2011).

Gene expression profiling studies have also been determined by studying the genes *AtPDR8, AtPDR12, AtATM3, AtPCR1, MPK4* and *MPK6* in HMs stressed plants under the influence of ISA. Lee et al. (2005) and Kim et al. (2006, 2007) revealed that tolerance of *Arabidopsis* to HM stressors might be related to *AtPDR8* and *AtPDR12* that function as metal ion export pumps to exclude HM (Fe, Cd and Pb) from the cytoplasm of the plant tissues. However, exogenous application of ISA might act as an extracellular signalling agent to regulate all the parameters of plant growth and via induction of tolerance mechanisms in plants. This is mainly achieved by initiating the biosynthesis of various enzymes including the activators of gene expressions related to Cd, Pb and Fe tolerance (Hien et al. 2000; Ma et al. 2010; Aftab et al. 2011). Also, Thapa et al. ( 2012), Song et al. (2004), and Kim et al. (2006) reported that, under the adverse impact of HMs, many plants stimulate detoxification processes through effective cellular mechanisms, such as chelation of HMs in the cytosol by peptides (phytochelatins) for the repair of stress‐damaged essential proteins. It is also accompanied by decreased uptake or efflux pumping in plasma membrane or through compartmentalization of metals inside the vacuole by *AtATM3, AtPDR12, AtPDR8,* and *ZNT1* (tonoplast‐located transporters), respectively. It also stimulates the synthesis of mitogen-activated protein kinases (MAPKs), which increases intracellular protein activity via phosphorylation and changes the expression levels of genes and protein synthesis (Smekalova et al. 2014). A study reported the modulation of expression levels of *Hordeum vulgare* growth and stress- related genes after alginate treatment (Yang et al. 2020). Although growth depend on cell division, differentiation, and auxin-mediated regulation of transcriptional auxin response factors (Guilfoyle and Hagen 2007). The upregulation in the expression of alginate-mediated gene responses *ARF3* and *RF17* revealed that they may express the seedling-related genes downstream and promote the growth of plants. *MAPK* genes encoding *MAPK* pathway also transmit upstream signal to activate the cascade for amplification towards downstream molecular responses via protein phosphorylation responsible for plant growth and other hormonal signal transducing pathways (Zhang et al. 2018). The modulation in the gene expression of these genes in the present study is due to their induced transcript levels along with the higher rate of translational processes and protein synthesis.

## Conclusion

The data in the present research clearly indicated that treatment with foliar sprays of ISA (75 mg/l) resulted in enhanced tolerance of mung bean plants against different HMs stresses. ISA induced photosynthetic activity and modulated the concentrations of MDA, proline, total phenols, and expression of genes encoding for stress tolerance (*AtPDR8/AtPDR12*) compared to controls. Therefore, the present investigations confirmed that there is a possibility to mitigate stress levels from plants along with improving their growth and yield attributes by using ISA. However, such study involving ISA-mediated growth and productivities of *V. radiata* plants have been conducted for the first time in this present investigation. The observations from this study may enable the researchers to optimize the ISA as they are cheap, renewable, environment friendly agent, non-harmful, and available in nature for stimulation of productivity as well as quality of plants. Nevertheless, further studies and investigations are essential to comprehend the associated mechanisms of ISA for stress alleviation and growth promotion in plants.
